# Endoscopic vacuum therapy in the upper gastrointestinal tract

**DOI:** 10.3389/fgstr.2024.1502682

**Published:** 2025-01-17

**Authors:** Kirsten Boonstra, Lisanne M. D. Pattynama, Yasmijn J. van Herwaarden, Wietse J. Eshuis, Roos E. Pouw

**Affiliations:** ^1^ Radboud University Medical Center, Department of Gastroenterology and Hepatology, Nijmegen, Netherlands; ^2^ Amsterdam UMC location Vrije Universiteit Amsterdam, Department of Gastroenterology and Hepatology, Amsterdam, Netherlands; ^3^ Amsterdam UMC location University of Amsterdam, Department of Surgery, Amsterdam, Netherlands; ^4^ Amsterdam Gastroenterology Endocrinology Metabolism, Amsterdam, Netherlands; ^5^ Cancer Treatment and Quality of Life, Cancer Center Amsterdam, Amsterdam, Netherlands

**Keywords:** endoscopic vacuum therapy, upper GI endoscopy, upper GI surgery, gastrointestinal perforation, Boerhaave syndrome

## Abstract

Endoscopic vacuum therapy (EVT) is a promising and versatile intervention for managing transmural defects in the upper gastrointestinal tract. Despite challenges, EVT exhibits great efficacy and safety, emphasizing the need for standardized protocols and evidence-based practices. We provide an overview, including mechanism, indications, types of EVT devices and complications, guiding clinicians in decision-making. Common challenges in EVT are highlighted, facilitating adequate implementation of EVT and helping to avoid common mistakes in daily practice.

## Endoscopic vacuum therapy

In the last decade, endoscopic vacuum therapy (EVT) has become an established treatment for upper gastrointestinal anastomotic leakage after surgery, iatrogenic perforations and Boerhaave syndrome. (1–3) Wedemeyer et al. first described the successful treatment of two patients with EVT for anastomotic leakage after esophagectomy in 2008. (4) Since then, implementation of and research on EVT in the upper gastrointestinal tract has been increasing, due to excellent success rates and only few adverse events. (5) Based on the principles of vacuum wound therapy, a sponge attached to a catheter is placed over the defect or in the adjacent cavity and negative pressure is applied, draining fluid and stimulating perfusion and secondary wound healing.

In the beginning, surgeons and endoscopists fabricated these draining catheters themselves using a tube and a very thin double-layered open-pore drainage film. (6) Subsequently, commercially available devices for the upper gastrointestinal tract have been developed, e.g., the EsoSPONGE (Braun B. Melsungen, Germany), a polyurethane sponge of 50mm in length and 13mm in diameter, and the VACStent (MICRO-TECH Europe GmbH, Düsseldorf, Germany), a nitinol-covered stent, with a polyurethane sponge attached to its outer surface and a suction catheter. This treatment combines the advantage of negative pressure wound therapy and sealing of the defect. The negative pressure prevents dislocation, and the stent facilitates oral intake.

EVT is increasingly being adopted in medical centers worldwide. Given the challenges associated with its implementation and utilization, sharing experiences can enhance understanding of the treatment and facilitate its effective implementation.

## Indications

There are three primary indications for EVT in the upper gastrointestinal tract: 1) anastomotic leakage following upper gastrointestinal surgery, 2) esophageal perforations resulting from iatrogenic causes, and 3) Boerhaave syndrome. Management of these defects is not standardized and includes conservative endoscopic and surgical options.

There are several contraindications for the use of EVT: 1) a defect that is larger than the sponge-part of the EVT device, 2) a defect too close to the upper esophageal sphincter, as proximity to the upper esophageal sphincter can lead to patient discomfort and difficulty in removing the device, and 3) proximity to a large blood vessel. A large cavity size is a relative contraindication, especially for the VACStent, since this device can only be applied intraluminal, and the cavity may then not be drained adequately.

Specifically for iatrogenic esophageal perforations, EVT can be used as a rescue therapy. For iatrogenic perforations arising during endoscopic therapeutic procedures or small perforations from foreign body ingestion, immediate endoscopic closure with through the scope clips (TTSC) is first line treatment. (3) When the perforation is too large or complex for immediate closure or closure fails, EVT is a good second option (7).

## Technique

EVT procedures should be performed under deep propofol sedation or general anesthesia, to allow for adequate assessment of the defect and introduction and placement of the device.

The defect and extraluminal cavity are measured and cleaned. Suction is applied to check if a cavity collapses. Endoluminal placement of a sponge or VACStent is suitable if the adjacent cavity is clean and collapsing. If necessary, a sponge can be trimmed to fit intracavitary. A sponge is placed using an overtube or grasping forceps and the VACStent is placed over a wire and deployed under direct endoscopic vision with a commonly used distal release system. The VACStent can also be placed and deployed under fluoroscopy guidance, depending on the preference of the endoscopist.

The vacuum tube is guided from the oral cavity to the nose and fixed with a plaster. Continuous vacuum therapy is applied ranging from -50 mmHg to -125 mmHg.

Intracavitary EVT is indicated in case of a large and/or contaminated cavity.

When using the intracavitary technique, it is crucial to leave a portion of the cavity unfilled with sponge material to allow for gradual reduction in size. For deep cavities this means the intracavitary sponge needs to be gradually placed more proximal with every sponge exchange to allow the distal part to collapse and adhere. It is advisable to leave part of the intra-cavitary sponge visible in the lumen of the esophagus, to prevent it from being enclosed in the cavity after granulation occurs. If the cavity is absent or small, intraluminal EVT can be used. The need for intracavitary treatment depends on multiple factors, such as defect and cavity size, degree of contamination, available resources and experience of the endoscopist.

## Efficacy

The vast majority of EVT studies include patients with anastomotic leakage after upper gastrointestinal surgery. Pooled data of (retrospective) case series show a high technical success rate of 97.1% and clinical success rate of 89.4%. (5) For anastomotic leakage, EVT seems to be superior to conventional endoscopic stent placement. (8–11) For other indications, especially iatrogenic perforations, no specific prospective data is available comparing stent and EVT.

A prospective case series of the first ten patients treated with a VACStent in a large tertiary referral center showed a success rate of 100%, requiring a median of 5 (IQR 3–12) EVT-related endoscopies with a treatment course of median 18 (IQR 12–59) days (12).

Analysis of pooled data from three prospective cohorts, where 92 VACStents were placed in 50 patients for esophageal leaks, showed a cure rate of 76% (38 out of 50 patients) (13).

## Safety

The reported adverse event (AE) rate in case series is low. In our first published study of 38 patients, two severe AEs occurred during EVT treatment (5%, 95%CI 1%–18%): a tracheoesophageal fistula, resulting in a re-operation with repair of the bronchial defect, gastric conduit resection (revealing unexpected carcinoma at histopathological examination) and cervical esophagostomy; and an iatrogenic defect expansion owing to friction of the overtube during a sponge exchange, after which re-operation with re-do anastomosis was performed. Two incidents occurred (5%, 95%CI 1%–18%): an esophageal ulcer due to the suction catheter and a minor hemorrhage during sponge removal (14).

This corresponds with literature, as sponge dislocation is the most common adverse event. (10, 11) The development of fistulae to the trachea, bronchi, or major vessels has been documented as an adverse event associated with EVT. (15, 16) The causal relationship of EVT and the occurrence of these severe adverse events is not known, as fistulae are a feared complication of anastomotic leakage with or without EVT. Nonetheless, despite its rarity, awareness of this risk is crucial when employing EVT.

Furthermore, EVT may result in fibrotic stenosis following defect closure, necessitating endoscopic dilation (8).

## Lessons learned

Undergoing a learning curve is an inevitable part of implementing a new technique in clinical practice. Sharing lessons learned can help colleagues shortening these learning curves and improve safety for our patients.

For example, introducing the sponge with a grasping forceps, instead of using an overtube, may prevent tearing and expansion of the defect. Sponges can safely be removed from the intraluminal position once a week and intracavitary every 3-4 days. In our experience, during the initial stage of implementation, a relatively high number of sponge ruptures occurred. A distal attachment cap placed on the endoscope helps to carefully separate the sponge from the mucosa. After all sides of the sponge have been loosened, the sponge can be easily removed, without needing to firmly pull it. (14) In case of the VACStent, a tapered distal attachment cap helps to maneuver between the stent and the esophageal wall. Secondly, switching off the vacuum pump several hours before removal of the VACStent facilitates easier removal. An important disadvantage of the VACStent is difficult removal near the upper esophageal sphincter. Placing the upper flange of the stent at least 3 centimeters below the upper sphincter is warranted.

In our practice, vacuum settings have gradually increased, starting cautiously at -50 mmHg intracavitary pressure. However, vacuum pressures up to -125 mmHg have been observed to be safe.

In our experience, when the sponge is removed after 3-7 days to prevent ingrowth, it can be reused if it is not damaged and can be cleaned adequately. This saves the need for a whole new sponge kit and is easy to implement as a sustainability measure. After the sponge is separated from the mucosa using a distal attachment cap, the sponge is moved from the defect to the gastric conduit using a grasping forceps. The tube is flushed and the sponge part is cleaned inside the gastric conduit using endoscopic flushing and pressure from the cap. After the sponge is cleaned it is replaced at the defect site using the grasping forceps.

Lastly, given the involvement of multiple departments and factors influencing success rates, multidisciplinary, patient-tailored decision-making is essential in EVT. Investing in education and establishing clear protocols can enhance multidisciplinary collaboration and help to anticipate logistical challenges (https://www.evt-academy.com).

## Future perspectives

Future research should focus on the best indications and techniques for EVT, as there are no clear universal protocols yet. With this better understanding of EVT, success rates may increase. Additionally, we expect the therapeutic range of EVT to expand in the coming years. Case series show promising results for closing duodenal defects with EVT. (17, 18) With global obesity rates on the rise, leakages after bariatric surgery are a growing indication for EVT (19).

Furthermore, the pre-emptive use of EVT may help prevent anastomotic leakage after upper gastrointestinal surgery (20).

## Data Availability

The original contributions presented in the study are included in the article/supplementary material. Further inquiries can be directed to the corresponding author.
